# Design of plasmonic cavities

**DOI:** 10.1186/s40580-014-0008-4

**Published:** 2014-03-07

**Authors:** Soon-Hong Kwon, You-Shin No, Hong-Gyu Park

**Affiliations:** 1 Department of Physics, Chung-Ang University, Seoul, 156-756 Republic of Korea; 2 Department of Physics, Korea University, Seoul, 136-701 Republic of Korea

## Abstract

In this review paper, we introduce the unique optical properties of high-quality, fully three-dimensional, subwavelength-scale plasmonic cavities. Surface-plasmon-polaritons excited at dielectric-metal interfaces are strongly confined in such cavities. The field profiles of plasmonic modes, their temperature-dependent quality factors, and subwavelength mode volumes are calculated and analyzed systematically using three-dimensional finite-difference time-domain simulations. Reasonable design of high-quality plasmonic cavities opens an opportunity to demonstrate novel plasmonic lasers enabling the further miniaturization of coherent light sources for use in ultra-compact photonic integrated circuits.

## Introduction

Plasmonic cavities are particularly attractive for nano-scale photonic applications as their physical sizes can be smaller than the diffraction limit of light [1–9], whereas the dimensions of conventional dielectric cavities such as photonic crystals [10–12], microdisks [13–15], nanowires [16–18] and metal-cladding cavities [19–22] are limited by wavelength. The resonant wavelengths of the surface-plasmon-polaritons (SPPs) excited at the dielectric-metal interface can be shorter than the wavelengths in dielectric cavities. Therefore, SPPs can be confined in a subwavelength volume. Various high-quality (Q) plasmonic cavities were successfully demonstrated by reducing metallic absorption [8], and provided strong optical feedback for lasing as well as an accessible collection of light emission [3,4]. Lasing using plasmonic cavities could be a significant step toward an ultimate miniaturized coherent light source for nano-scale photonic integrated circuits. In this review paper, we will introduce four recently reported high-Q plasmonic cavities for strong SPP confinement and theoretically investigate their optical properties using three-dimensional (3D) finite-difference time-domain (FDTD) methods.

## Review

### Simulation Method

In our FDTD simulations, silver, which was used for the metal component in the plasmonic cavities we investigate, was modeled with a Drude model [23,24]: ε(ω) = ε_∞_ − ω_p_^2^ / (ω^2^ + iγω). The Drude model fits the experimentally determined dielectric function of silver. Dielectric functions in the visible spectral range (400 to 800 nm) and the near-infrared spectral range (800 to 2000 nm) were used to fit the Drude model, respectively [25]. In the visible spectral range, the background dielectric constant ε_∞_, the plasma frequency ω_p_, and the collision frequency γ at room temperature are 4.1, 1.4 × 10^16^ s^-1^, and 4.2 × 10^13^ s^-1^, respectively. In the near-infrared spectral range, the background dielectric constant ε_∞_, the plasma frequency ω_p_, and the collision frequency γ at room temperature are 3.1, 1.4 × 10^16^ s^-1^, and 3.1 × 10^13^ s^-1^, respectively.

In order to investigate the details of photon loss channels in plasmonic cavities, we calculated corresponding Q factors separately [2–4,8,9]. The Q factor of a SPP mode is determined by contributions from both the intrinsic metal loss and optical radiation loss into free space. As the optical radiation loss is reduced, the total Q factor of a SPP mode approaches the minimum value allowed by the intrinsic properties of the metal used, the metal-loss-limited Q factor. The Q factor due to optical radiation loss can be calculated with γ = 0. To represent the dielectric function of silver at low temperature, the damping collision frequency γ, was scaled by a factor of the room-temperature conductivity divided by the low-temperature conductivity [2–5,8,9,19]. The total Q factor was calculated from the time decay of the energy of a cavity mode [10,11]. To calculate mode volume and the confinement factor, we used the effective refractive index of the metal, ε_eff_ = d(ωε(ω)) / dω = ε_∞_ + (ω_p_ / ω)^2^, where ω is the resonant frequency of a SPP cavity mode [26]. The mode volume, V, is defined as the ratio of the total electric field energy density of the mode to the peak energy density [10,11,26]:

$$ V=\frac{1}{ \max \left[\frac{1}{2}{\varepsilon}_{eff}{\left|E\left(\overrightarrow{r}\right)\right|}^2\right]}\underset{ all}{\int \int \int}\frac{1}{2}{\varepsilon}_{eff}{\left|E\left(\overrightarrow{r}\right)\right|}^2{d}^3\overrightarrow{r} $$

### Plasmonic cavities

#### Dielectric-core/metal-shell nanowire plasmonic cavity

**Figure 1:**
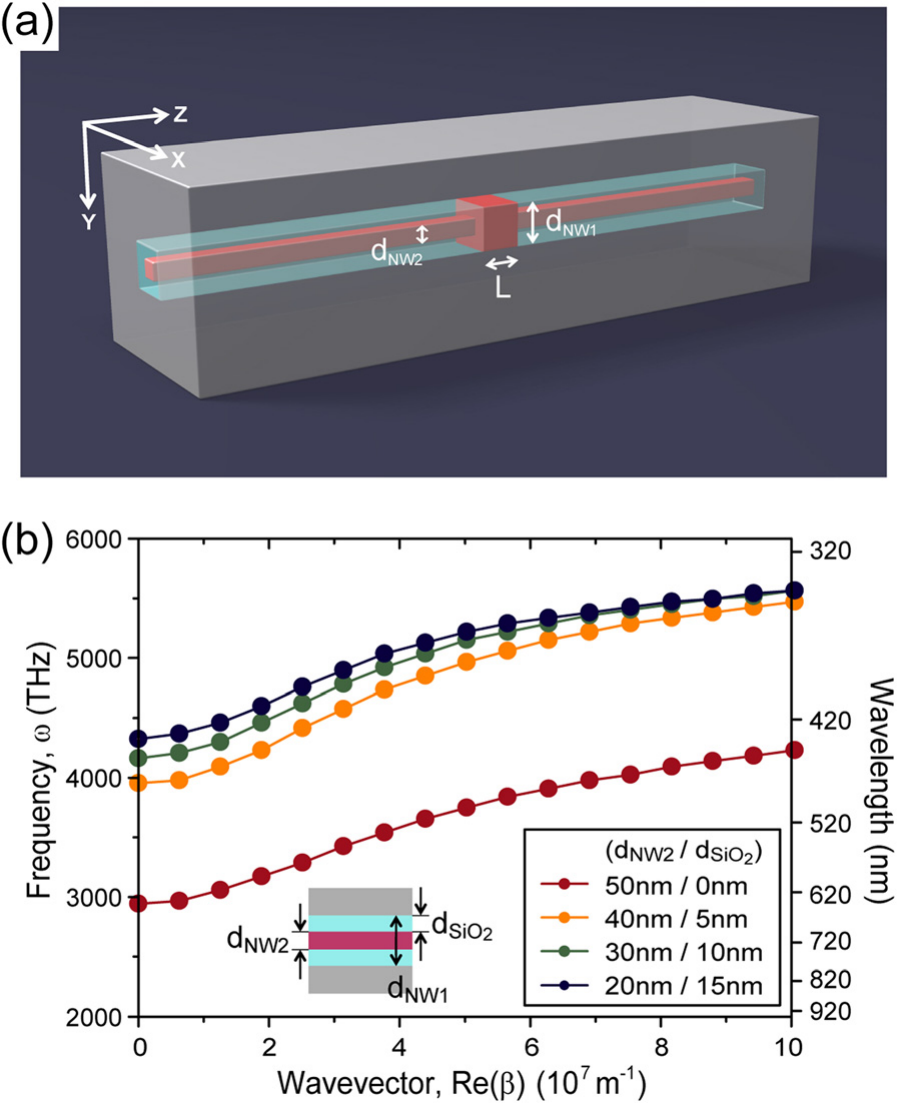
**3D confinement of SPPs in a deep subwavelength plasmonic cavity. (a)** Schematic diagram of the plasmonic cavity. d_NW1_, d_NW2_, and L represent the nanowire diameter of the cavity region, the diameter of the high-index core, and the cavity length, respectively. **(b)** Calculated dispersion curves of the fundamental transverse SPP modes in the square cross-sectional, infinitely long waveguide with high-index-core/low-index-shell/metal-shell structure. Refractive indices are 2.6 and 1.5 for the high-index core and the low-index shell, respectively. Silver is used as the metal shell. Inset: d_NW1_ is fixed to 50 nm, and d_NW2_, and d_SiO2_ are the thicknesses of the high-index core, and the low-index shell, respectively. Adapted from [2].

A plasmonic cavity based on a dielectric-core/metal-shell nanowire structure can enable deep subwavelength confinement of SPPs in all three spatial dimensions (Figure 1(a)) [2]. This plasmonic cavity is formed by introducing an axial heterostructure along the nanowire axis in a 2D plasmonic waveguide of metal-coated dielectric nanowire. The SPPs excited at the interface between the high-index dielectric nanowire core and the metal shell are confined in a cavity region with core diameter d_NW1_ and length L. Two high-index-core/low-index-shell/metal-shell nanowire structures with a smaller core diameter d_NW2_ effectively form a plasmonic mirror, sandwiching the cavity region. The refractive indices of the high-index core and low-index shell are 2.6 (e.g. CdS or InGaN in the visible wavelength [27,28]) and 1.5 (SiO_2_), respectively, and the metal shell is made of silver.

The longitudinal confinement of SPPs in this cavity can be understood from the dispersion curves of the fundamental transverse plasmonic-guided mode in a square cross-sectional, infinitely long waveguide with a high-index-core/low-index-shell/metal-shell structure (inset of Figure 1(b)). The total diameter of the nanowire including the high-index core and the low-index shell, d_NW1_, is fixed at 50 nm. The frequency of the SPP mode approaches a non-zero minimum as the wavevector approaches zero, which is the cutoff frequency. SPP modes with lower frequencies than this cutoff frequency cannot propagate along the waveguide. This cutoff frequency, a unique property of the proposed 2D plasmonic waveguide [29], depends significantly on the presence of a low-index shell d_SiO2_ (Figure 1(b)), explaining the large frequency gap between waveguides with and without it. We note that this frequency gap prevents the coupling of SPP modes excited in a waveguide lacking a low-index shell with those excited in a waveguide with a low-index shell. Therefore, three-dimensional (3D) confinement of SPPs is achieved in the region without the low-index shell, without modifying the metallic structure.

**Figure 2:**
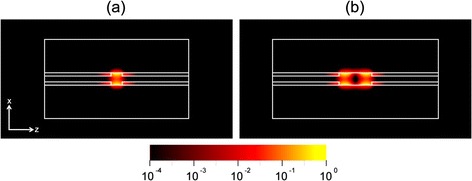
**Electric field intensity (log E** ^**2**^ **) mode profiles in the plasmonic cavities with (a) L = 40 nm and (b) L = 120 nm.** The number of electric field antinodes, m, the resonant wavelengths, λ, and the Q factors for both cavities are as follows: **(a)** m = 1, λ = 533 nm, Q = 36100, **(b)** m = 2, λ = 529 nm, Q = 38100. Q factors are calculated at 20 K. Adapted from [2].

In order to investigate the confined SPP modes, we calculated the cavity modes for the cavities with L values of 40 and 120 nm, respectively. The electric field intensity (log E^2^) mode profiles show that SPPs are strongly confined in the cavities without significant scattering (Figure 2). Consequently, strong suppression of optical radiation loss allows these cavities to achieve high Q factors (greater than 36000) approaching the metal-loss-limited value. In this case, the Q factors were calculated at the low temperature of 20 K. At this temperature, we could assume a reduced absorption loss value for silver. We note that the proposed plasmonic cavity exhibits two unique properties: the deep subwavelength-scale confinement of SPPs and extremely low optical radiation loss. The physical dimensions of the cavity are 50 × 50 × 40 nm^3^ at a wavelength of 533 nm. In addition, the longitudinal SPP cavity modes can be identified by the number of the electric field antinodes, m, in a similar fashion to a conventional Fabry-Perot cavity. As L increases, the value m of an excited SPP mode increases. For example, in the cavity with L = 40 nm, the longitudinal mode of m = 1 is observed in the wavelength of interest (Figure 2(a)).

**Figure 3:**
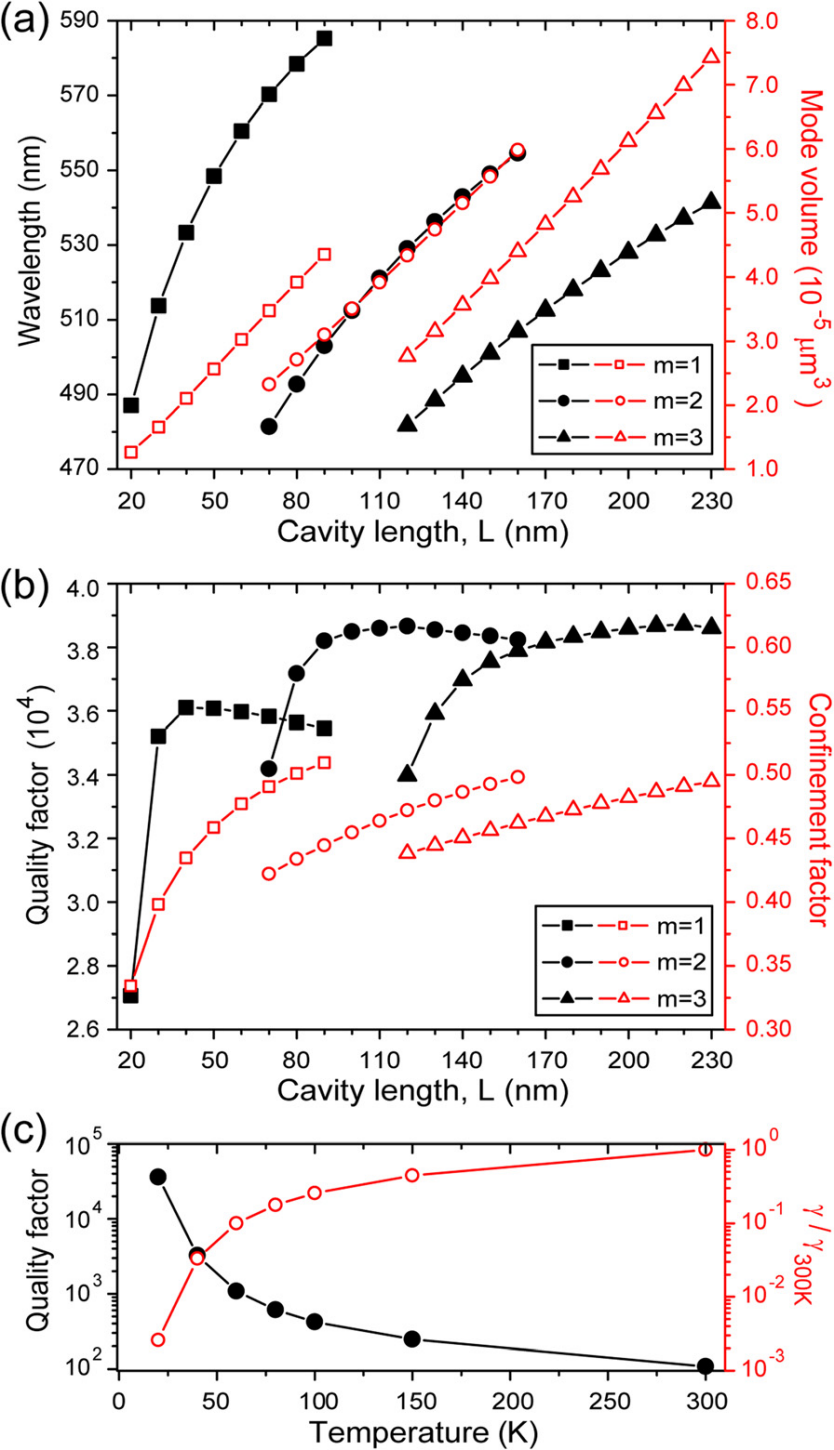
**The optical properties of the SPP modes with m = 1, 2, and 3. (a)** The resonant wavelengths and mode volumes are plotted as a function of L. An extremely small mode volume on the order of 10^-5^ μm^3^ results. **(b)** The Q factors and confinement factors are plotted as a function of L at low temperature (20 K). **(c)** The Q factor of the SPP mode of m = 1 for the cavity with L = 40 nm is plotted as a function of temperature. Adapted from [2].

Figure 3 shows the optical characteristics of the plasmonic cavity, such as the resonant wavelengths, mode volumes, Q factors, and confinement factors. The resonant wavelength can be tuned readily over a wide spectral range by varying L and thus can be well adjusted to the emission spectrum of a dielectric nanowire (left axis, Figure 3(a)). In addition, extremely small mode volumes on the order of 10^-5^ μm^3^ were calculated (right axis, Figure 3(a)). These are ∼ 100 times smaller than those of the smallest dielectric cavities so far reported [10–12,19–22]. The physical limit of an optical cavity’s size, which is the diffraction limit of light, is overcome in the plasmonic cavity. For example, in a cavity with L = 40 nm and m = 1 (Figure 2(a)), the mode volume was calculated to be ∼ 1/50 (λ/2n_NW_)^3^, where n_NW_ is 2.6. Next, Q factors of the SPP cavities were calculated at a temperature of 20 K (Figure 3(b)). The high Q factor of ∼ 38000 approaches the metal-loss-limited value because optical radiation loss for this cavity is negligible. Indeed, the optical Q factor is estimated at an extremely high value of 3.2 × 10^6^ with zero collision frequency. As L and the resonant wavelength decrease, the Q factor tends to decrease in a cavity with a fixed m. Also, the high Q factor and subwavelength-scale mode volume of this SPP mode yield an extremely high λ^3^Q/V value of ∼ 2.6 × 10^8^. This value is comparable to the best values of dielectric cavities such as photonic crystal or microdisk cavities [11,12,19,30]. In addition, the plasmonic cavity exhibits a large confinement factor, which is here defined as the ratio of the energy confined in the dielectric nanowire core of the cavity to the total energy of the cavity mode. It was calculated to be > ~ 0.45 (right axis, Figure 3(b)).

To investigate the effect of metallic absorption loss on Q factors, we plotted Q factors for the SPP mode with m = 1 in a 40-nm-long cavity as a function of temperature (Figure 3(c)). Since lowering temperature reduces the absorption loss of the metal and increases the metal-loss-limited Q factor [23,31], high-Q SPP modes can be obtained at low temperatures. The metal-loss-limited Q factor depends predominantly on the damping collision frequency, γ. The SPP mode’s Q factor increases exponentially with decreasing temperature due to the dramatic reduction of collision frequency. Even at 80 K, a high Q factor of ∼ 600 was obtained. The Q factor’s inverse-proportional relationship to γ shows that the Q factor is limited by metal absorption loss, and that the optical loss of the SPP cavity mode is negligible.

#### Nanorod plasmonic cavity using cutoff mirror mechanism

**Figure 4:**
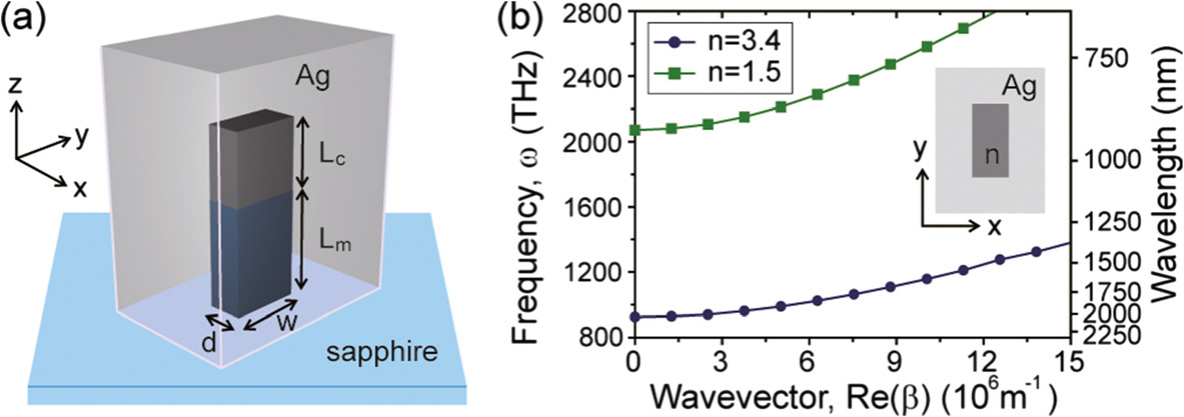
**Plasmonic cavity with a high-index (top)/low-index (bottom) dielectric nanorod covered by silver. (a)** Schematic diagram of the cavity. The cavity is placed on a sapphire substrate. **(b)** Calculated dispersion curves of the fundamental SPP mode for the core refractive index of 3.4 (blue) and 1.5 (green), with w = 200 nm and d = 100 nm. Adapted from [9].

Maximizing the ratio between Q factor and mode volume V, i.e., Q/V, is an effective mean to increase light-matter interaction. Instead of increasing the Q factor, which is metal-loss-limited in the plasmonic cavity, we reduced the size of the plasmonic cavity by using high-index/low-index dielectric nanorods covered with silver (Figure 4(a)) [9]. The lengths and refractive indices of the high- (low-) index dielectric nanorods are L_c_ (L_m_) and 3.4 (1.5), respectively. Along the z-axis, the SPP mode is confined at the high-index dielectric-silver interface due to the large frequency gap between the SPP mode excited at the high-index dielectric-silver interface and the SPP mode excited at the low-index dielectric-silver interface [2]. Since the top of the nanorod is covered with silver, a subwavelength SPP cavity mode is confined along the opposite z-axis, with an extremely small mode volume. In rectangular cross-sectional dielectric waveguides covered with silver, dispersion curves calculated for the fundamental SPP waveguide modes show how the SPP confinement is built (Figure 4(b)). In the waveguides, the rectangular cross-section is defined by w = 200 nm and d = 100 nm, and the refractive indices (n) of dielectric cores are 3.4 (circles) and 1.5 (squares), respectively. In Figure 4(b), the cutoff frequencies in these 2D plasmonic waveguides strongly depend on the refractive index of the dielectric core of the waveguide, n. The cutoff frequency increases (926 to 2072 THz) with decreasing n (3.4 to 1.5). The structural parameters of the waveguides, such as w and d, also affect the cutoff frequencies.

**Figure 5:**
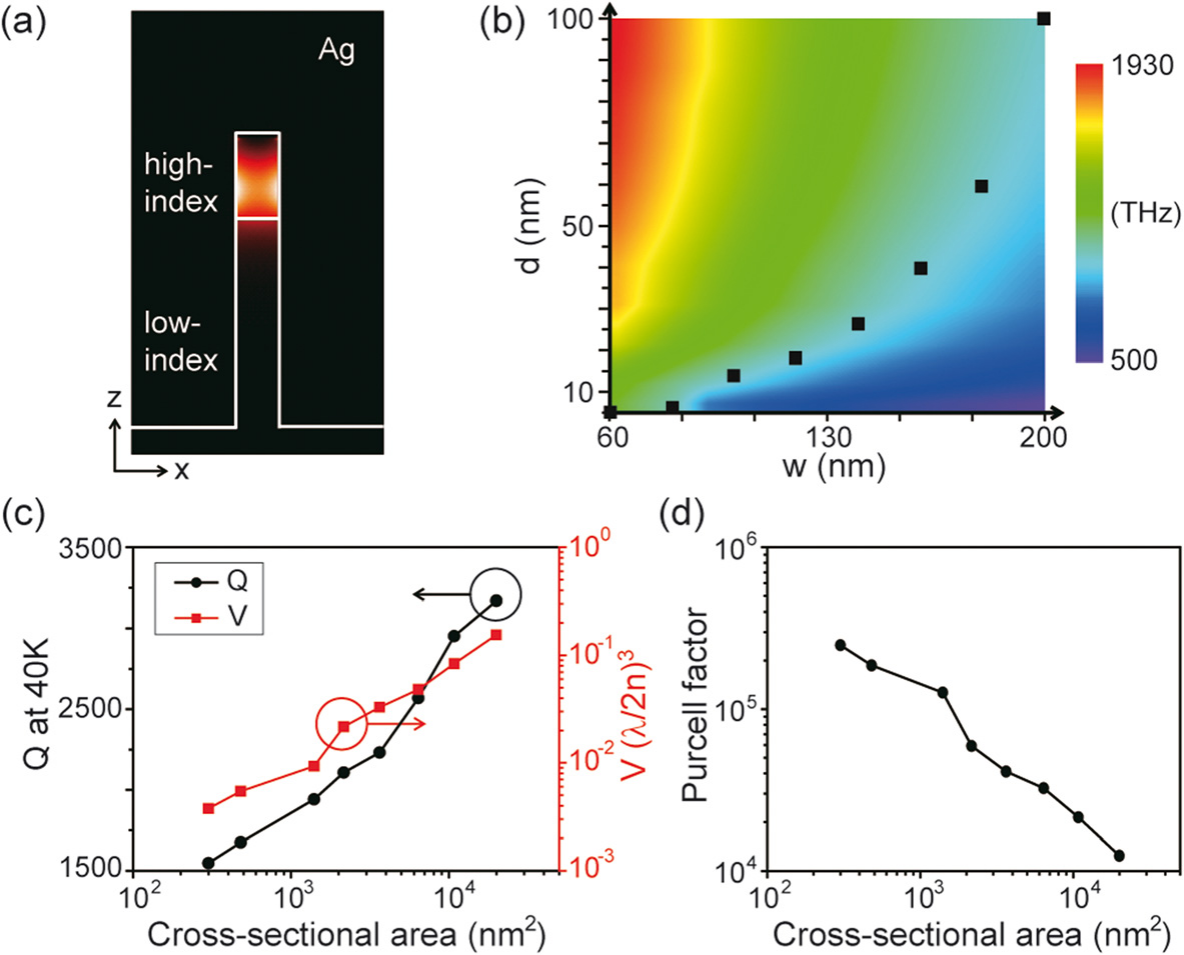
**Optical properties of the high-index/low-index dielectric nanorod SPP cavity. (a)** Electric field intensity profile of the SPP mode in the cavity. L_c_ = 200 nm, w = 200 nm, and d = 100 nm. **(b)** Cutoff frequency color map of the high-index dielectric core-silver shell SPP waveguide plotted as a function of w and d . The cutoff frequency decreases with increasing w and decreasing d. **(c)** Q factors at 40 K (black) and mode volumes (red) plotted as a function of the cross-sectional area, w × d. (w, d) ranges from (200 nm, 100 nm) to (60 nm, 5 nm). **(d)** Purcell factor plotted as a function of the cross-sectional area. Adapted from [9].

To further reduce the cavity size while maintaining the resonant wavelength, we performed systematic FDTD simulations to obtain the cutoff frequencies of high-index dielectric-silver waveguide modes for various structural parameters (Figure 5). Here, a dipole emitter was used to excite a SPP mode, which was placed 1 nm away from the sidewall of the silver surface. The SPPs are strongly confined at the high-index dielectric-silver interface (Figure 5(a)). In the color map of the cutoff frequencies plotted as a function of w and d, red (top left) and violet (bottom right) indicate higher and lower frequencies, respectively (Figure 5(b)). The resonant frequency of the SPP mode in the various cavities can be estimated based on this map, because the frequency of the cavity mode is slightly higher than the cutoff frequency. For example, if one wanted to design a cavity which has a resonant wavelength of 1550 nm, the cavities with dimensions w and d corresponding to the black dots in Figure 5(b) can be used, where the cutoff frequencies are 1550 nm.

Next, in a nanorod plasmonic cavity with L_c_ = 200 nm, the Q factors at 40 K and mode volumes were investigated as a function of the cross-sectional area of the cavity, w × d (Figure 5(c)). Since solid-state cavity quantum electrodynamics (QED) experiments studying light-matter interactions such as those involving a single photon source and strong coupling are usually performed in the temperature range from 0 to 40 K [26,32,33], the Q factor at 40 K suggests a minimum for the metal-loss-limited Q factor. The dimensional parameters, w and d, (represented as black dots in Figure 5(b) were chosen so that the resonant wavelengths remain at 1550 nm. The mode volumes (red) decrease significantly from 0.15 to 0.0038 (λ/2n)^3^ with decreasing cross-sectional area from 2 × 10^4^ to 3 × 10^2^ nm^2^, where the mode volume decreases by a factor of 40. The Q factors (black), however, decrease only slightly, from 3200 to 1500. This discrepancy is due to higher metallic absorption in the smaller cavity. We note that the mode volumes decrease much more sharply than Q factors do with decreasing physical cavity size.

The extremely small mode volume of the proposed cavity enables it to have large Purcell factors even though metallic absorption loss always occurs in a plasmonic cavity. In Figure 5(d), the Purcell factors, F, were calculated as a function of the cross-sectional area using the following equation [32]:

$$ F=\frac{3}{4{\pi}^2}{\left(\frac{\lambda }{n}\right)}^3\left(\frac{Q}{V}\right) $$

Here, an emitter was assumed to be placed at the modal field maximum to calculate the maximum enhancement of Purcell factor. A large Purcell factor, greater than 2 × 10^5^, was obtained for a cavity with dimensions 60 × 5 × 200 nm^3^ (Figure 5(d)). Spontaneous emission from this cavity can be enhanced considerably due to this large Purcell factor. Even if a tenfold drop in Q factor is considered at room temperature the Purcell factor remains large, ∼2 × 10^4^, owing to the tiny mode volume of 0.0038 (λ/2n)^3^.

#### Room-temperature channel-waveguide plasmonic cavity

**Figure 6:**
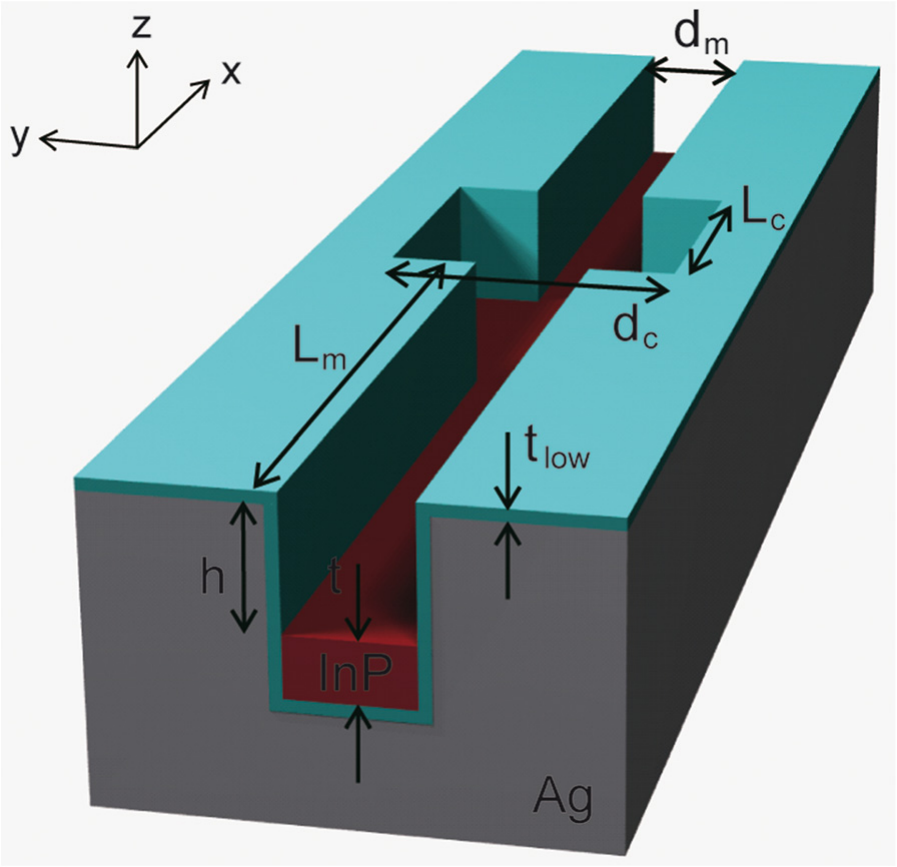
**Channel-waveguide plasmonic cavity.** SPP modes are confined in the cavity region with a width of d_c_ and length of L_c_. Low-index and high-index layers are indicated with cyan and red colors. Adapted from [8].

In order to reduce metallic absorption loss and increase Q factor at room temperature, a channel-waveguide plasmonic cavity is proposed by combining two silver-air channel waveguides of different widths and introducing a low-index layer on the sides and bottom walls of the silver (Figure 6) [8]. The widths of the wide and narrow waveguides are d_c_ and d_m_, respectively. A high-index dielectric slab is added on the bottom. The thicknesses of the low-index layer and high-index slabs are t_low_ and t, respectively. In this cavity, SPPs excited at the bottom silver surface are strongly confined within the wide waveguide with length L_c_. The narrow-channel waveguides at both sides of the cavity prevent the propagation of SPP modes along the y-axis.

**Figure 7:**
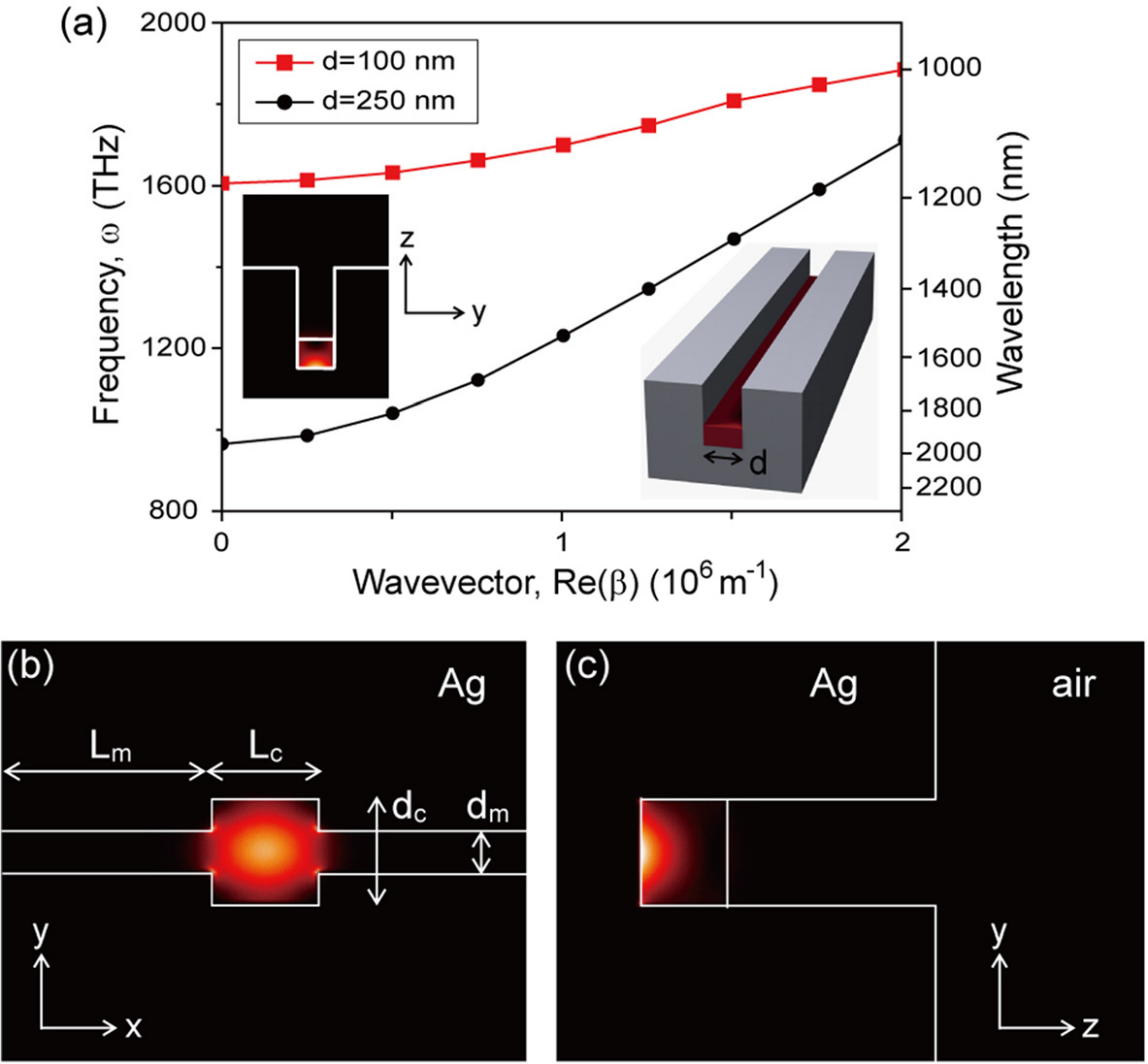
**Field confinement in the channel-waveguide plasmonic cavity. (a)** Dispersion curves of the SPP modes for the waveguides of different widths, 250 nm (black) and 100 nm (red). The right inset illustrates a channel waveguide consisting of a high-index dielectric slab and an air slot with a rectangular cross-section. The left inset shows the electric field intensity profile of the SPP waveguide mode. **(b)** Top and **(c)** cross-sectional views of the electric field intensity profile of the SPP cavity mode. d_c_ = 250 nm, L_c_ = 250 nm, d_m_ = 100 nm, L_m_ = 500 nm, t = 200 nm, and h = 500 nm. Adapted from [8].

In a channel waveguide without a low-index layer, the SPP mode is confined at the interface of the silver and the high-index slab (left inset, Figure 7(a)). The dispersion curves significantly depend on the waveguide width, d. The cutoff frequency, appearing due to the finite waveguide width, decreases with increasing d [2,34]. The cutoff frequency of the waveguide with d = 100 nm is 1606 THz, while it is 965 THz for d = 250 nm. Therefore, the SPP mode excited in the waveguide with d = 250 nm cannot propagate into the waveguide with d = 100 nm and the narrower waveguide can act as a mode-gap mirror as long as the frequency remains in the frequency mode gap between 965 and 1606 THz. The mode-gap mirror can be used to form a channel-waveguide plasmonic cavity that consists of a wider waveguide between two narrower waveguides.

3D FDTD simulations show that a SPP cavity mode is confined efficiently in the channel-waveguide plasmonic cavity (Figure 7(b)). L_c_ and h are large enough (L_c_ > 250 nm and h > 200 nm) to achieve strong SPP confinement. In the top and side views of the electric field intensity profiles, the SPP mode is confined at the cavity’s bottom dielectric-silver interface (Figure 7(c)). Along the y-axis of the waveguide, the SPP cavity mode with a resonant wavelength of 1550 nm (1216 THz) is confined within the wide waveguide region with dimensions d_c_ × L_c_ (250 nm × 250 nm) by the mode gap (965–1606 THz). The mode is confined along the x-axis by metallic mirrors formed with the side walls. Both of these confinement mechanisms, the mode gap and the use of metal mirrors, can be used to demonstrate the possibility of a subwavelength-scale 3D plasmonic cavity.

Losses in the metallic cavity can again be divided into optical loss and metallic absorption loss. In the channel-waveguide plasmonic cavity, radiation into free space is strongly suppressed. Therefore, optical loss can be assumed to be negligible and the cavity achieves strong optical feedback. An ultra-high optical Q factor of 1.2 × 10^9^ was calculated by neglecting metallic absorption in the proposed channel-waveguide plasmonic cavity. The mode volume was estimated to be extremely small, λ^3^/10000 or 0.0040 (λ/n)^3^, where λ and n are the wavelength in free space and the refractive index of the high-index slab, respectively. As temperature increases, metallic absorption loss also increases, dominating the cavity’s total losses [3,4]. The Q factor of the plasmonic cavity significantly decreases from 1.2 × 10^9^ at 0 K to 125 at room temperature due to increased metallic absorption. The resonant wavelength and mode volume remained nearly constant across all temperatures.

**Figure 8:**
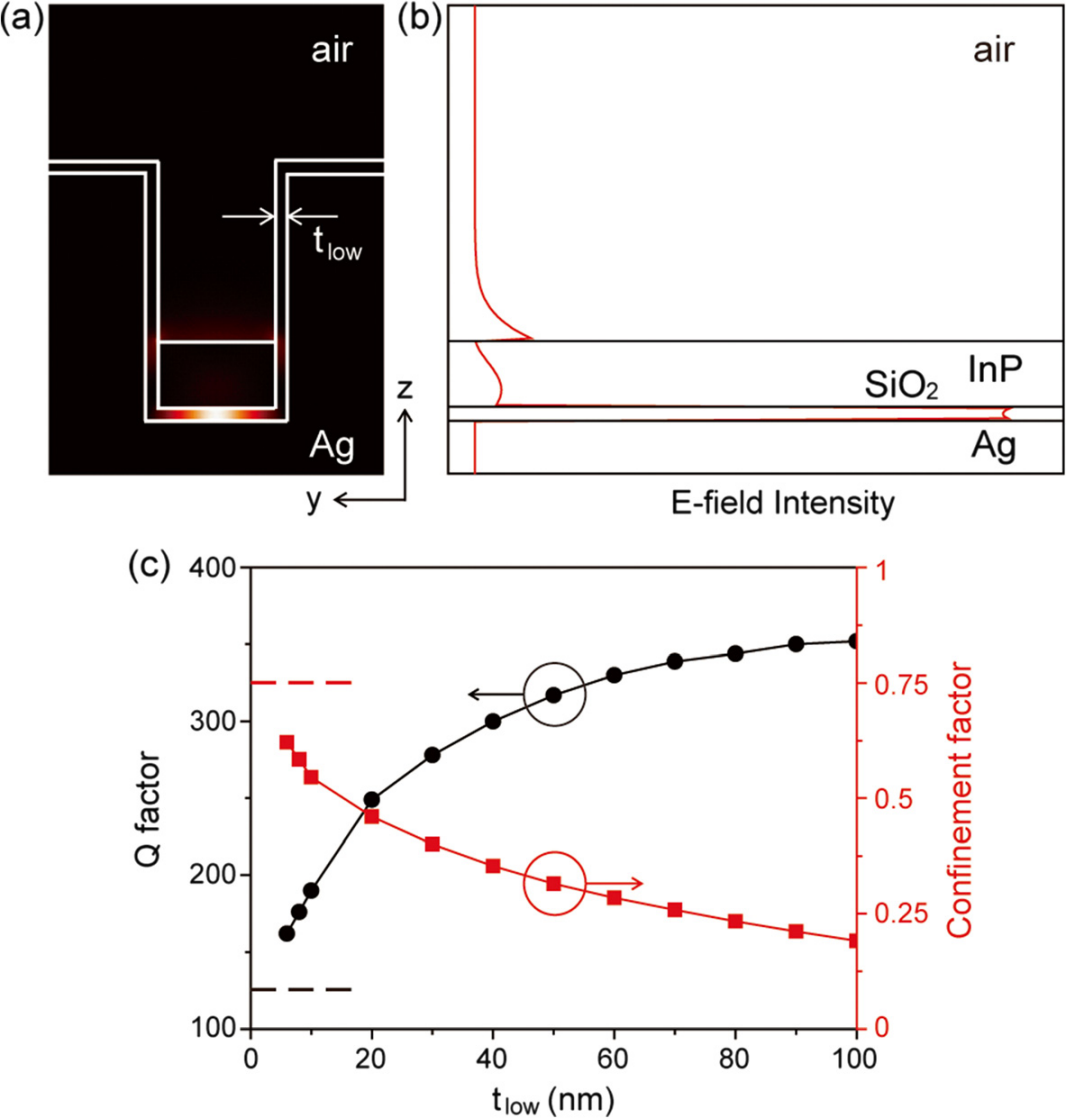
**Plasmonic cavity with a low index layer of thickness t**_**low**_ **. (a)** Cross-sectional electric field intensity profile.** (b)** Electric field intensity distribution along the z-axis at the center of the cavity. **(c)** Q factors (black) and confinement factors (red) at room temperature plotted as functions of t_low_. Dotted lines indicate the Q and confinement factors of the cavity without a low-index layer. Adapted from [8].

In this case, high metallic absorption of the SPP mode is unavoidable due to the electric field overlapping with metal. Therefore, to achieve high Q at room temperature, it is necessary to minimize the amount of field energy of the SPP mode extending into metal. An efficient way to reduce metallic loss is to introduce a low-index layer at the dielectric-metal interface [35]. In the channel-waveguide plasmonic cavity, a low-index layer with a refractive index of 1.5 (e.g. SiO_2_) was introduced at the silver interface (Figure 7(a)). When t_low_ = 40 nm, d_c_ = 350 nm, and L_c_ = 350 nm, a SPP cavity mode with a resonance wavelength of 1550 nm is strongly confined in the cavity region because of the mode gap mentioned above. Figures 8(a) and (b) show that most of the electric field intensity is strongly confined in the low-index layer, which should decrease metallic absorption loss. For this plasmonic cavity, the Q factor at room temperature is 300, which is 2.5 times larger than that of 125 for the cavity without a low index layer. The introduction of a low-index layer slightly increases the mode volume to λ^3^/1000 or 0.040 (λ/n)^3^, where n is the refractive index of the high-index slab.

To examine the performance of the proposed plasmonic cavity with a low-index layer, we calculated the Q (black) and confinement (red) factors at room temperature as a function of the thickness of the low-index layer, t_low_ (Figure 8(c)). Here, the confinement factor is defined by the ratio between the energy in the high-index slab and total energy of the cavity mode. The Q factor increases significantly with increasing t_low_ because the amount of mode energy overlapping into the silver decreases (Figure 8(b)). The Q factor increases up to 350, which is larger than the Q factor of 125 for the cavity without the low-index layer. On the other hand, introducing of the low-index layer decreases the confinement factor with increasing t_low_ because the energy in the low-index layer increases. Since both high Q and high confinement factors are desirable for lasing, the thickness of the low-index layer need to be optimized for the room-temperature operation of plasmonic lasers.

#### Nanodisk/nanopan plasmonic cavity

**Figure 9:**
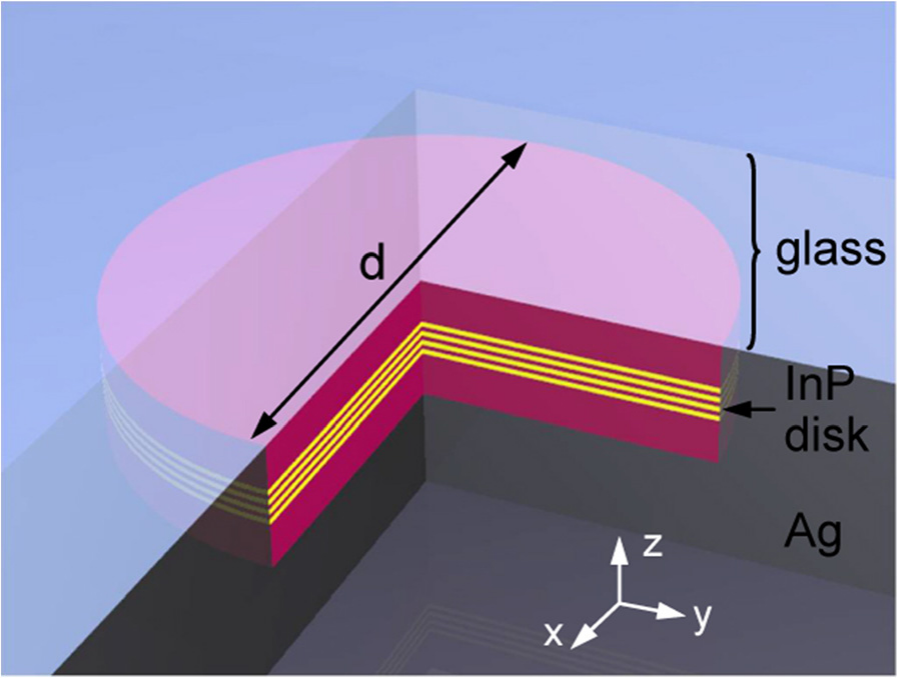
**Schematic diagram of the nanodisk/nanopan plasmonic cavity.** Adapted from [3].

We finally examine a nanodisk/nanopan plasmonic cavity. This cavity consists of an InP nanodisk with a radius of r and a silver nanopan covering the bottom and sidewall surfaces of the nanodisk (Figure 9) [3,4]. Various SPP modes are excited at the disk-nanopan interfaces in conjunction with the suppression of optical modes. In particular, high-Q whispering-gallery (WG) modes, which are typically observed in a conventional disk cavity, appear as SPP modes in the nanodisk/nanopan structure. In addition, the silver nanopan surrounding the sidewall of the disk suppresses radial optical loss and enables the excitation of radial SPP modes, which are barely observed in a conventional disk-shaped cavity. In fact, using this nanodisk/nanopan structure, plasmonic lasing operation was experimentally demonstrated at 10 K [3,4]. Optical pumping and photoluminescence (PL) collection were performed through the glass substrate bonded to the nanodisk (Figure 9).

**Figure 10:**
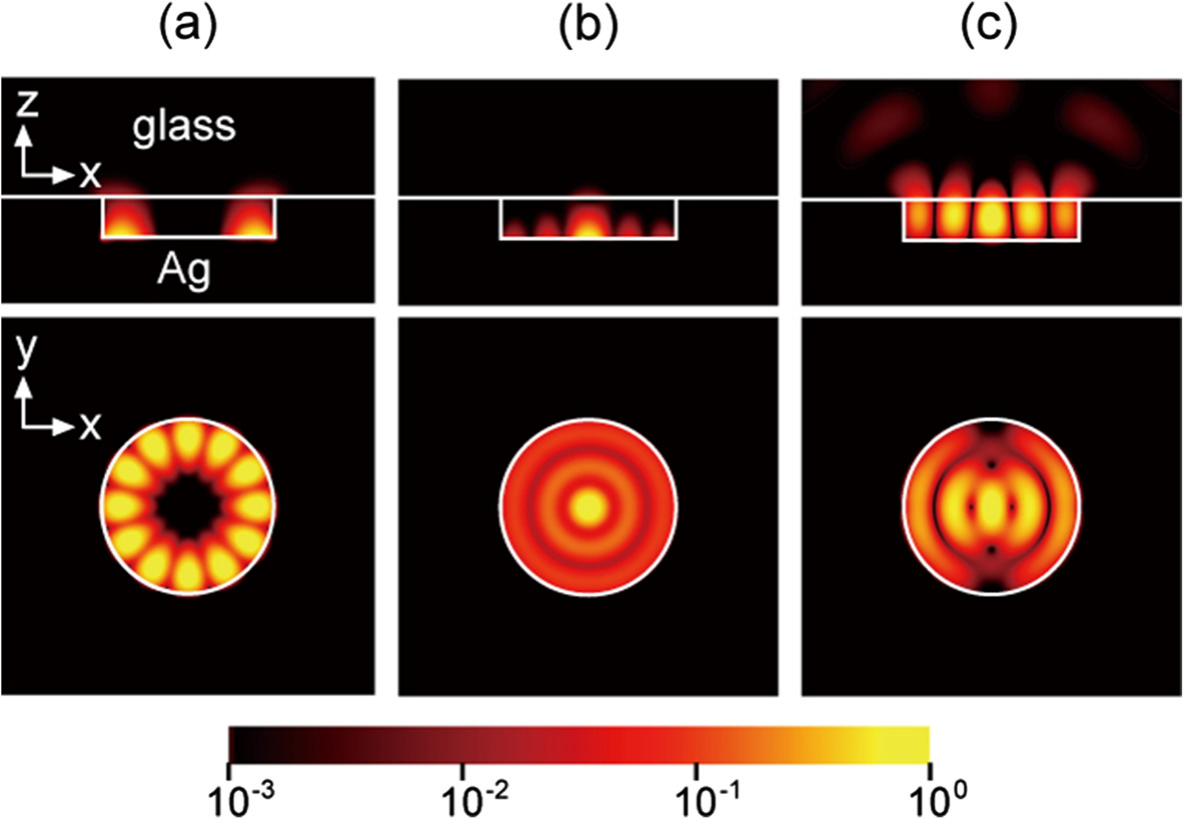
**Side (upper panel) and top (lower panel) views of the electric field intensity profiles (log-scale) of (a) TM-like WG SPP, (b) TM-like radial SPP, and (c) dipole optical modes.** Adapted from [3].

In order to understand the SPP modes excited in this nanodisk/nanopan structure, all possible cavity modes were modeled using FDTD simulation [3,4]. In the simulation, the refractive indices of the InP and glass were 3.2 and 1.45, respectively. Representative resonant modes are shown for a nanodisk/nanopan structure with a nanodisk radius of 500 nm in Figure 10. Among three modes, two SPP modes (transverse-magnetic-like (TM-like) radial and TM-like WG) have an electric field maximum at the disk-silver interface, whereas one optical mode (a dipole mode) has an electric field maximum at the center of the dielectric disk. The TM-like WG SPP and dipole optical modes are doubly degenerate but the TM-like radial SPP is non-degenerate. The TM-like WG and radial SPP modes have subwavelength mode volumes of 0.56 (λ/2n)^3^ and 0.65 (λ/2n)^3^, respectively, which are smaller than the diffraction limit of light. On the other hand, the mode volume of the optical dipole mode, 2.7 (λ/2n)^3^, is three times larger than this limit.

The WG SPP modes derive from the conventional WG optical modes [13–15,30]. For example, the dominant electric field of conventional TM-like WG optical modes excited in a nanodisk without a silver nanopan is directed outward from the bottom surface of the disk. Introducing the silver nanopan converts these optical modes into the TM-like WG SPP modes. On the other hand, the transverse-electric-like (TE-like) WG optical modes are converted into the TE-like WG SPP modes because all of their dominant electric fields point in a perpendicular direction to the sidewall of the disk.

**Figure 11:**
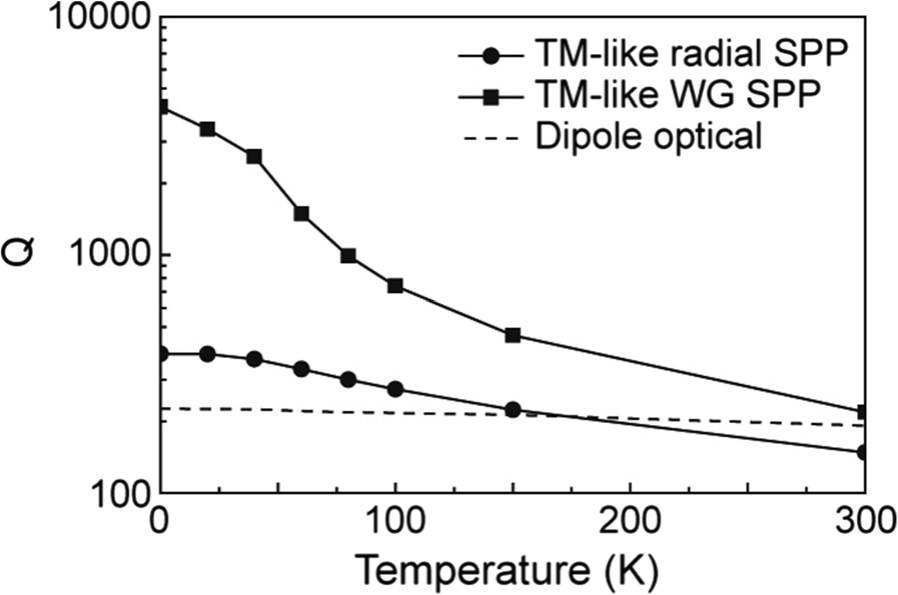
**Q factors of TM-like WG/radial SPP modes and dipole optical mode plotted as a function of temperature.** The disk radii are all 500 nm in this simulation. Adapted from [3].

The Q factors of three excited modes in the nanodisk/nanopan cavity were calculated as a function of temperature using a FDTD simulation (Figure 11). The TM-like WG and radial SPP modes show significant temperature-dependence in their Q factors. At low temperature, the Q factors of the SPP modes are higher than that of the optical mode (dipole mode) due to low metallic absorption loss and relatively low optical radiation loss, however, they seriously deteriorate with increasing temperature and absorption loss [2–4]. In contrast, the Q factors of the optical mode remained nearly constant over the calculated range of temperatures. This result agrees well with the consideration that the SPP modes’ electric fields extend considerably into the metal, whereas the optical mode has relatively small overlap with the metal nanopan. Indeed, strong temperature-dependent optical loss of SPP modes is a major factor distinguishing SPP modes from conventional optical modes.

## Conclusion

In summary, we investigated the optical properties of four subwavelength-scale plasmonic cavities: a dielectric-core/metal-shell nanowire plasmonic cavity, a nanorod plasmonic cavity using a cutoff mirror mechanism, a channel-waveguide plasmonic cavity, and a nanodisk/nanopan plasmonic cavity. Experimental demonstrations of deep subwavelength-scale photonic devices such as single photon sources, plasmonic lasers, optical memory devices, and ultrasmall biochemical sensors can be expected based on these theoretical plasmonic cavity structures with ultrasmall cavity sizes. In particular, plasmonic lasers may prove to be promising coherent light sources, as they enable the miniaturization of nanophotonic devices as well as the ultra-compact integration of photonic systems requiring minimal thermal overhead.

## References

[1] Kuttge M, de Abajo FJG, Polman A (2010). Nano Lett..

[2] Seo MK, Kwon SH, Ee HS, Park HG (2009). Nano Lett..

[3] Kwon SH, Kang JH, Seassal C, Kim SK, Regreny P, Lee YH, Lieber CM, Park HG (2010). Nano Lett..

[4] Kwon SH, Kang JH, Kim SK, Park HG (2011). IEEE J. Quantum Electron..

[5] Hill MT, Marell M, Leong ESP, Smalbrugge B, Zhu Y, Sun M, van Veldhoven PJ, Geluk EJ, Karouta F, Oei Y-S, Notzel R, Ning C-Z, Smit MK (2009). Opt. Express.

[6] Oulton RF, Sorger VJ, Zentgraf T, Ma R-M, Gladden C, Dai L, Bartal G, Zhang X (2009). Nature.

[7] Ma R-M, Oulton RF, Sorger VJ, Bartal G, Zhang X (2011). Nat. Mater..

[8] Kang JH, Park HG, Kwon SH (2011). Opt. Express.

[9] Kang JH, No YS, Kwon SH, Park HG (2011). Opt. Lett..

[10] Painter O, Lee RK, Scherer A, Yariv A, O’Brien JD, Dapkus PD, Kim I (1999). Science.

[11] Park HG, Kim SH, Kwon SH, Ju YG, Yang JK, Baek JH, Kim SB, Lee YH (2004). Science.

[12] Nozaki K, Kita S, Baba T (2007). Opt. Express.

[13] Liu L, Kumar R, Huybrechts K, Spuesens T, Roelkens G, Geluk E-J, de Vries T, Regreny P, Thourhout DV, Baets R, Morthier G (2010). Nat. Photonics.

[14] Song Q, Cao H, Ho ST, Solomon GS (2009). Appl. Phys. Lett..

[15] Kim YH, Kwon SH, Lee JM, Hwang MS, Kang JH, Park WI, Park HG (2012). Nat. Commun..

[16] Huang MH, Mao S, Feick H, Yan H, Wu Y, Kind H, Weber E, Russo R, Yang P (2001). Science.

[17] Duan X, Huang Y, Agarwal R, Lieber CM (2003). Nature.

[18] Seo MK, Yang JK, Jeong KY, Park HG, Qian F, Ee HS, No YS, Lee YH (2008). Nano Lett..

[19] Hill MT, Oei YS, Smalbrugge B, Zhu Y, de Vries T, van Veldhoven PJ, van Otten FWM, Eijkemans TJ, Turkiewicz JP, de Waardt H, Geluk EJ, Kwon SH, Lee YH, Nötzel R, Smit MK (2007). Nat. Photonics.

[20] Yu K, Lakhani A, Wu MC (2010). Opt. Express.

[21] Nezhad MP, Simic A, Bondarenko O, Slutsky B, Mizrahi A, Feng L, Lomakin V, Fainman Y (2010). Nat. Photonics.

[22] Lu CY, Chang SW, Chuang SL, Germann TD, Bimberg D (2010). Appl. Phys. Lett..

[23] Maier SA (2007). Plasmonics: Fundamentals and Applications; Springer.

[24] Taflove A, Hagness SC (2000). Computational Electrodynamics: The Finite-Difference Time-Domain Method.

[25] Johnson PB, Christy RW (1972). Phys. Rev. B.

[26] Gong YY, Vuckovic J (2007). Appl. Phys. Lett..

[27] Palik ED (1985). Handbook of Optical Constants of Solids.

[28] Fang Q, Li Y, Gradecak S, Park H-G, Dong Y, Wang ZL, Lieber CM (2008). Nat. Mater..

[29] Dionne JA, Lezec HJ, Atwater HA (2006). Nano Lett..

[30] Vahala K (2003). Nature.

[31] Min B, Ostby E, Sorger V, Ulin-Avila E, Yang L, Zhang LX, Vahala K (2009). Nature.

[32] Englund D, Fattal D, Waks E, Zhang B, Nakaoka T, Arakawa Y, Yamamoto Y, Vuckovic J (2005). Phys. Rev. Lett..

[33] Reithmaier JP, Sek G, Loffler A, Hofmann C, Kuhn S, Reitzenstein S, Keldysh LV, Kulakovskii VD, Reinecke TL, Forchel A (2004). Nature.

[34] Dionne JA, Sweatlock LA, Atwater HA, Polman A (2006). Phys. Rev. B.

[35] Oulton RF, Sorger VJ, Pile DFP, Genov DA, Zhang X (2008). Nat. Photonics.

